# Disseminated gonococcal infection secondary to a rare homozygous mutation resulting in complement factor I deficiency

**DOI:** 10.70962/jhi.20250088

**Published:** 2025-08-18

**Authors:** Marianna Almpani, Geoffrey Welch, Emma Vanderleeden, Jutamas Shaughnessy, Qadija Qadri, Doyle V. Ward, Juan M. Perez-Velazquez, Sanjay Ram, Jennifer P. Wang

**Affiliations:** 1 Division of Infectious Diseases and Immunology, Department of Medicine, UMass Chan Medical School, Worcester, MA, USA; 2 Department of Medicine, Diabetes Center of Excellence, UMass Chan Medical School, Worcester, MA, USA; 3 Department of Microbiology, UMass Chan Medical School, Worcester, MA, USA

## Abstract

A 34-year-old male presented with recurrent fever, arthralgia, and rash. He had two prior hospitalizations for suspected vasculitis and meningococcal meningitis at age 13 years. Positive blood cultures for *Neisseria gonorrhoeae* established the diagnosis of disseminated gonococcal infection. Complement studies showed low total (CH50) and alternative pathway (AP50) activity and low levels of all alternative and terminal complement proteins and the complement inhibitors factor H and factor I (FI), suggesting uninhibited complement activation and complement consumption. A homozygous nonsense mutation leading to a premature stop codon (p.Arg474*, or R456X) in *CFI* predicted complete FI deficiency, which was confirmed by immunoblotting. The patient’s serum contained IgG against reduction-modifiable protein and the lipoproteins H.8 and lipidated azurin, which are targets for blocking antibodies against gonococci and meningococci, respectively. This report underscores the importance of considering FI deficiency in patients with invasive neisserial infections, vasculitis-like manifestations, and consumptive complementopathy.

## Teaser

We report a case of disseminated gonococcal infection linked to complement factor I (FI) deficiency, with a prior vasculitis-like episode. FI deficiency should be suspected in the setting of complement consumption, characterized by low levels of multiple complement components.

## Introduction

Disseminated gonococcal infection (DGI) is a rare but serious complication of *Neisseria gonorrhoeae* infection. Invasion of the bloodstream by *N. gonorrhoeae* can lead to septic arthritis, polyarthralgia, tenosynovitis, rash, and less commonly meningitis or endocarditis (1, 2, 3). Deficiencies of components of the alternative and terminal complement pathways have long been implicated in increasing the risk for neisserial infections (4, 5). Here, we report a patient who presented with DGI and was found to have a rare homozygous mutation leading to complement factor I (FI) deficiency.

## Results

The patient is a 34-year-old Hispanic male who presented to the emergency department (ED) in March 2024 with fever, arthralgia, and rash. He reported 2 wk of low-grade fever, bilateral ankle arthralgia with swelling, and a rash over his back, both arms, and legs. He additionally reported poor appetite and weight loss of ∼40 pounds over the past 4 mo.

His past medical history included two similar episodes that required hospitalization, the first in April 2021 and subsequently in November 2023 for which infectious diseases workup included negative blood cultures and transesophageal echocardiogram, while immunologic workup was notable for positive c-antineutrophil cytoplasmic antibody (1:160), low C3c (34 mg/dl; normal reference range 82–185 mg/dl), normal C4c, and negative antinuclear antibody, myeloperoxidase, antiproteinase 3, rheumatoid factor, and human leukocyte antigen B27. At the time, he was suspected to have IgA vasculitis and was treated with prednisone that was tapered over several weeks. He had Poland syndrome, a condition characterized by the partial or complete absence of the pectoralis major muscle, possibly resulting from a vascular insult during early embryological stages (6); in this case, it manifested as right chest wall hypoplasia and right-hand malformations. He had meningococcal meningitis at age 13 years and a prior methicillin-resistant *Staphylococcus aureus* infection involving the groin at age 17 years. The patient had no known allergies and was not taking any medications. He denied smoking or using intravenous or other recreational drugs. He occasionally consumed alcohol. He did not have any tattoos. He denied any recent travel. He reported one female sexual partner with whom he had two children. He admitted facing housing instability. His family history was negative for recurrent infections, and his parents, two siblings, and two school-age children were alive and healthy.

**Figure 1.**
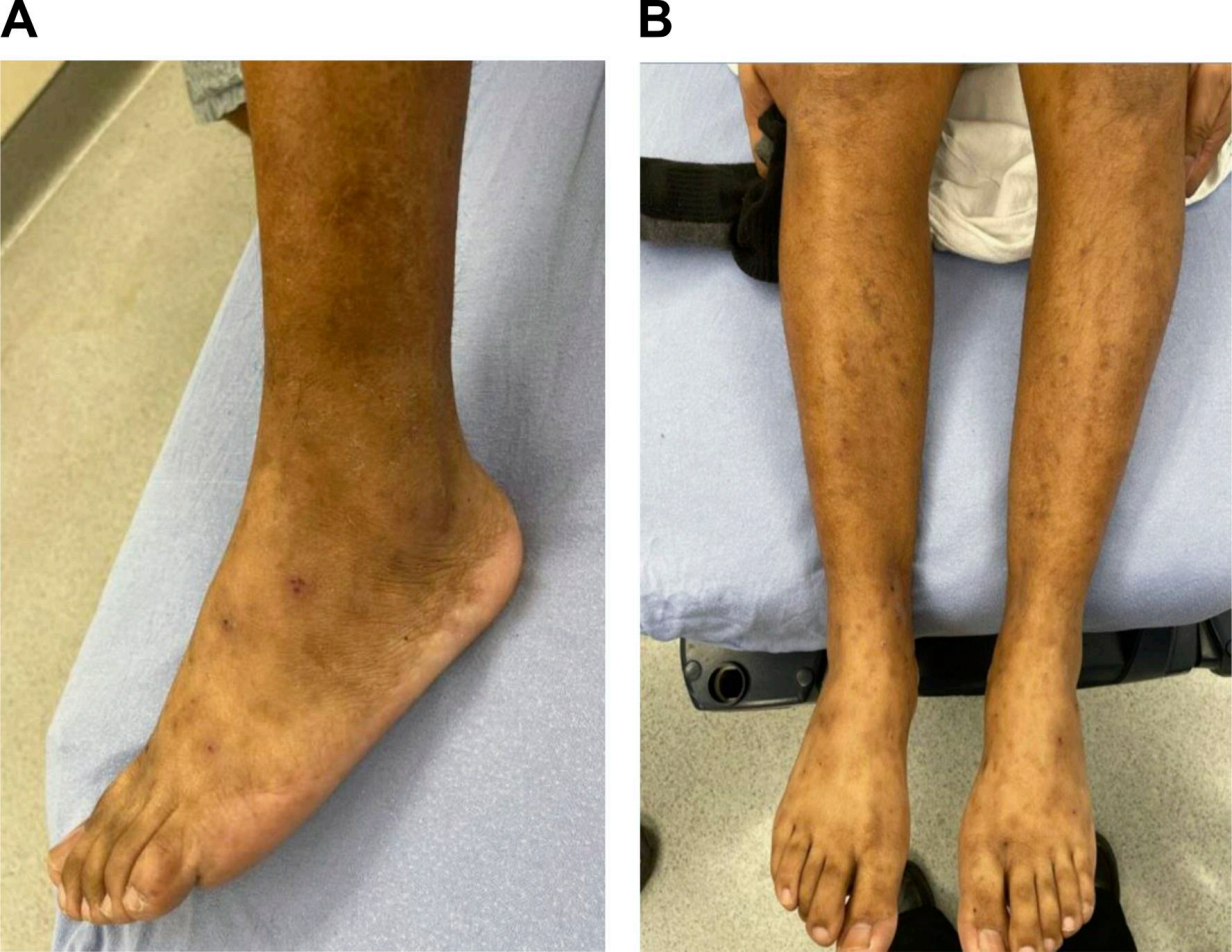
**Examination of the patient in March 2024. (A and B)** Admission photos show (A) lateral aspect of left foot and (B) bilateral lower extremities below the knees. **(A and B)** March 2024 admission photos of (A) lateral aspect of left foot and (B) bilateral lower extremities below knees.

**Figure 2.**
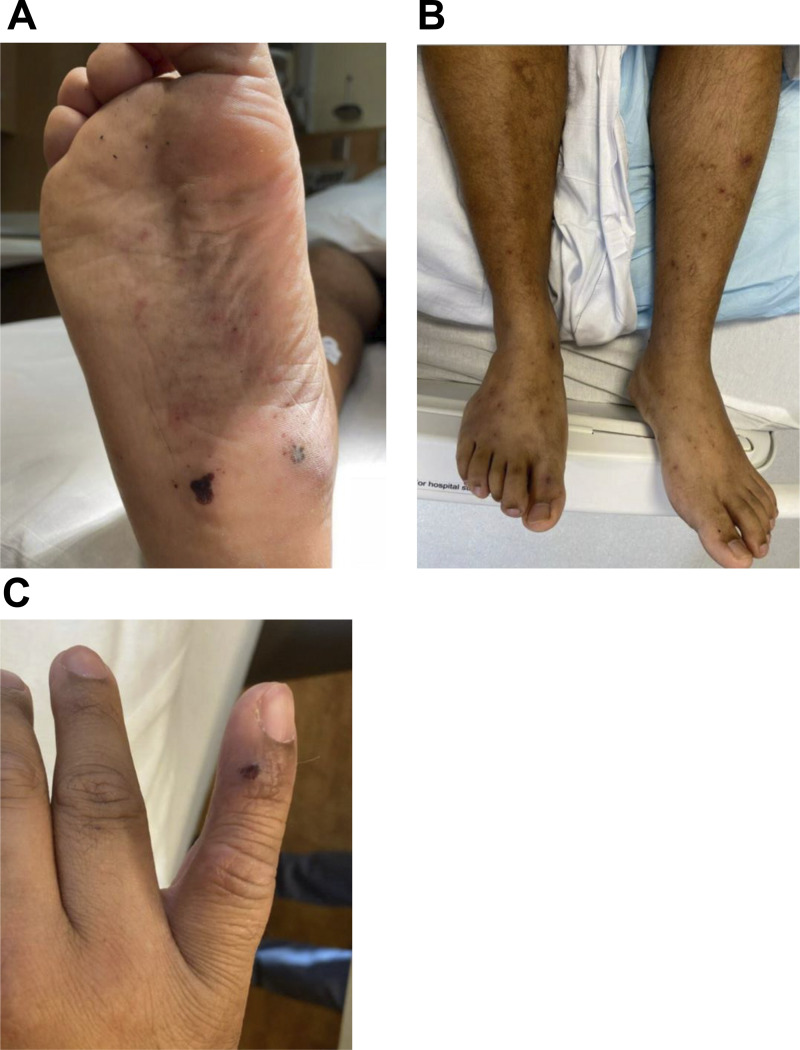
**Examination of the patient in April 2021 and in November 2023. (A and B)** Photos from April 2021 show (A) right foot purpuric rash and (B) bilateral lower extremity rash below the knees. **(C)** Photos from November 2023 show (C) left hand rash and finger swelling. **(A–C)** April 2021 photos of (A) right foot purpuric rash and (B) bilateral lower extremity rash below the knees, and November 2023 photo of (C) left-hand rash and finger swelling.

During this initial encounter in the ED, the peripheral white blood cell count was 17.9 × 10^3^/μl (83% neutrophils) and blood cultures were obtained. The patient was referred for a skin biopsy as an outpatient with planned corticosteroid initiation and was discharged home. However, the next day the blood cultures were reported to be positive and he was asked to return for further evaluation and admission to the inpatient medicine service. He was afebrile, the heart rate was 106 beats/min, the blood pressure was 115/81 mm/Hg, and the oxygen saturation on room air was 100%. Physical examination was notable for the absence of the right pectoralis muscle and hand deformity (baseline), as well as multiple scattered, nontender papules on the back and all four extremities, with evidence of postinflammatory hyperpigmentation and scarring (Fig. 1, A and B). These were overall consistent with images obtained from prior episodes, with purpuric lesions being particularly prominent in previous admissions (Fig. 2).

Laboratory studies from admission included a peripheral white blood cell count of 6.1 × 10^3^/μl, C-reactive protein of 199 mg/L, and erythrocyte sedimentation rate of 69 mm/h. His basic metabolic profile and liver function tests were unremarkable. His urinalysis was positive for protein (1+) and urobilinogen. Aerobic blood culture bottles from blood samples collected during the first ED visit, as well as newly obtained ones, were positive for gram-negative diplococci, later identified as *N*. *gonorrhoeae* sensitive to cefixime, cefoxitin, ceftriaxone, ciprofloxacin, and spectinomycin. Treponema pallidum antibodies, rapid plasma reagin, and fourth-generation HIV tests were negative. Urethral and throat swabs for *Chlamydia trachomatis* and *N*. *gonorrhoeae* RNA were negative. His immunoglobulin panel showed an elevated IgA of 511 mg/dl (reference range 47–310 mg/dl), while IgG and IgM levels were within normal limits. The patient was started on ceftriaxone 2 g intravenously daily. Blood cultures drawn 48 h after antibiotic initiation were negative, and transthoracic echocardiogram was unremarkable. His total complement (CH50) in a sample collected 48 h after antibiotic initiation was <10 U/ml (reference range 31–60 U/ml). He received a total of 7 days of intravenous ceftriaxone prior to discharge.

At the outpatient infectious diseases clinic follow-up visit 6 wk later, the patient reported complete resolution of symptoms. Given concern for complement deficiency, tetravalent meningococcal capsular conjugate (MenQuadfi), tetanus toxoid conjugate, and pneumococcal polysaccharide conjugate (PCV20) vaccines were administered. Laboratory tests collected at this visit included an alternate complement pathway activation test (AP50) that was low (<16%; normal level ≥31%), a complement protein profile performed at the Cincinnati Children’s Hospital Medical Center reference laboratory with results shown in Table 1, and genetic testing with the Complement Deficiency Disorders Panel offered by Invitae Corp. This revealed homozygosity for a nonsense mutation at codon 1420 (c.1420C>T) leading to a stop signal instead of translation to arginine (p.Arg474*, also referred to as variant R456X) in the serine protease domain of complement FI. The other 22 complement genes sequenced were unremarkable for genetic variants.

**Table 1.** Complement profile results for the patient

​	Result (L, low; H, high)	Reference range considered normal
C3	23.4 mg/dl (L)	71–150 mg/dl
C4	17.7 mg/dl	15.7–47 mg/dl
C1 esterase inhibitor	19.1 mg/dl (L)	21–39 mg/dl
C1q	8.9 mg/dl	7.5–20 mg/dl
C2	4.6 mg/dl (H)	1.9–3.6 mg/dl
C5	1.7 mg/dl (L)	2.8–6.8 mg/dl
C6	3.2 mg/dl (L)	4–6.9 mg/dl
C7	<1.4 mg/dl (L)	3.5–7.9 mg/dl
C8	2.8 mg/dl (L)	3–5.8 mg/dl
C9	1.3 mg/dl (L)	2.5–7.5 mg/dl
Factor B	<15 mg/dl (L)	19–50 mg/dl
FH	8 mg/dl (L)	15.8–37.5 mg/dl
FI	1.3 mg/dl (L)^a^	1.6–3.7 mg/dl
Properdin	7.9 μg/ml (L)	8.1–18.4 μg/ml
C4-binding protein	43.3% (L)	61–116%

a See explanation in text.

**Figure 3.**
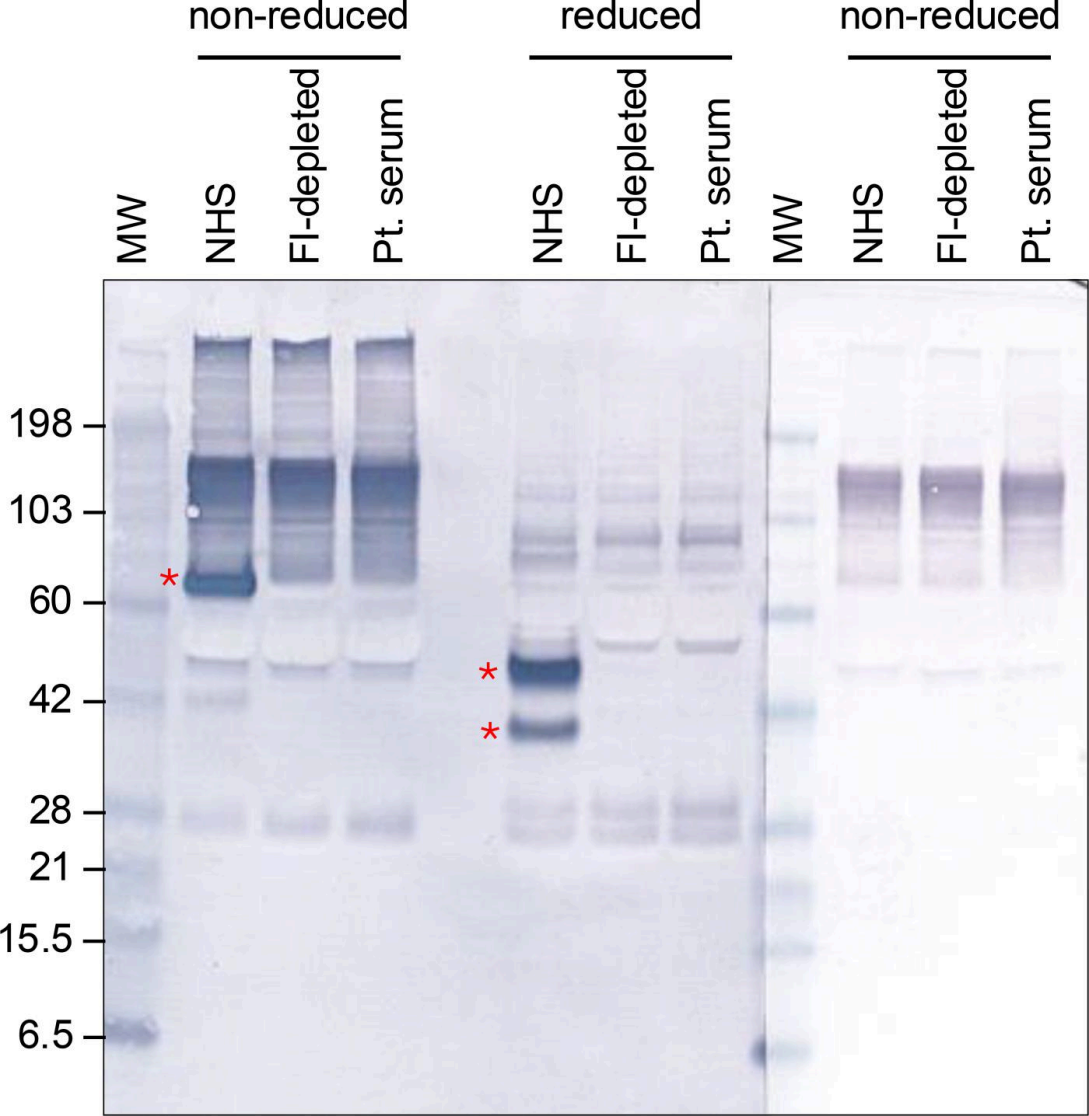
**Reactivity of FI-deficient patient’s serum with goat anti-human FI.** Proteins in NHS, FI-depleted serum, and the Pt. serum, all at a final dilution of 1:100 in 4× LDS sample buffer (either nonreduced or reduced with 10% ß-mercaptoethanol), were separated on a 4–12% Bis-Tris gel and probed with goat polyclonal anti-FI antibody followed by ALP-conjugated anti-goat IgG. Asterisks highlight detection of FI (combined heavy and light chains in nonreduced samples, heavy and light chains in reduced samples) in NHS and absence in FI-depleted serum and the patient’s serum. A third parallel section of the membrane (nonreduced samples) was probed with the secondary antibody (ALP-conjugated anti-goat IgG) only. All sections of the blot were exposed to substrate for the same length of time. MW, molecular weight marker (kDa); Pt. serum, patient’s serum. Source data are available for this figure: SourceData F3.

Given the discrepancy between the patient’s complement protein profile measured by nephelometry, which showed low but detectable FI, and the reported genetic mutation, which predicts the absence of FI (7), we tested the patient’s serum for FI by western blot. FI heavy (50 kDa) and light (38 kDa) chains were detected in normal human serum (NHS) but were absent from both the patient’s serum and commercially available FI-depleted serum (Fig. 3).

**Figure S1.**
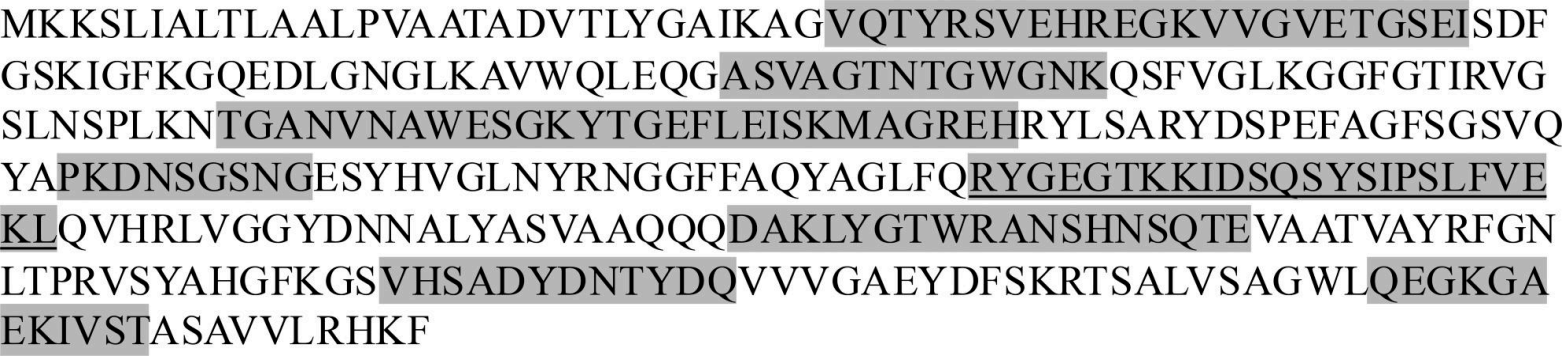
**PorB sequence of the patient’s *N. gonorrhoeae* isolate reveals class PorB.1B.** The PorB amino acid sequence of the patient’s isolate is shown with the eight surface-exposed loops highlighted in gray. A major difference between PorB.1B and PorB.1A lies in loop 5 (underlined); PorB.1A has a shorter loop 5 (usually ∼9 amino acids) than PorB.1B (8). The sequence shows 100% amino acid identity to the PorB (NEIS2020) allele 5325 (PorB.1B) in PubMLST.

**Figure 4.**
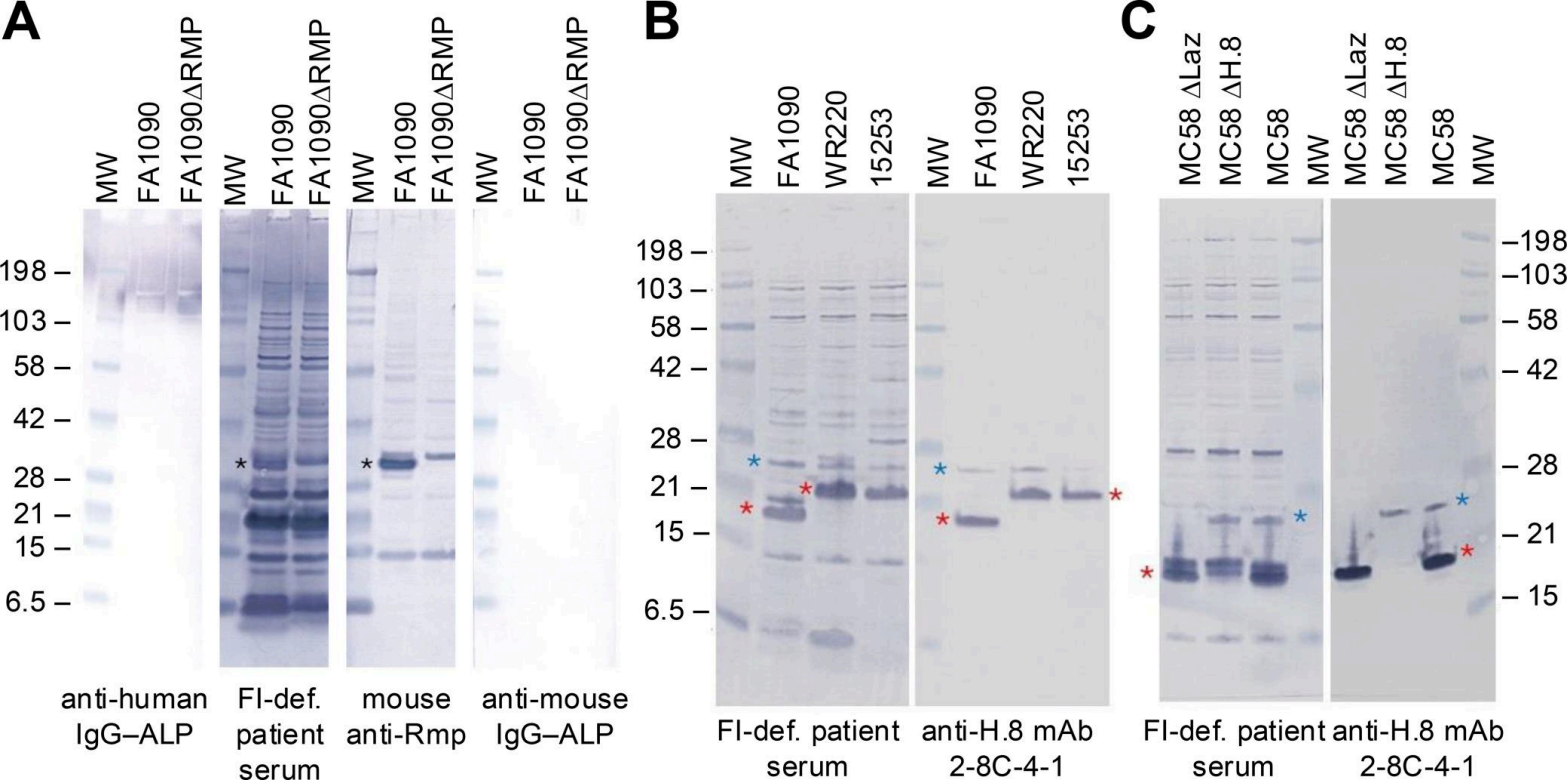
**Reactivity of IgG in FI-deficient patient’s serum with bacterial lysates of *N. gonorrhoeae*. (A)** Reactivity of the FI-def. patient’s serum against *N. gonorrhoeae* Rmp. Lysates prepared from *N. gonorrhoeae* strain FA1090 and its Rmp deletion mutant (FA1090 ΔRmp) were separated on a 4–12% Bis-Tris gel using MES SDS running buffer and transferred to a membrane by western blotting. The blot was divided into four sections and probed with the patient’s serum (1:500 dilution) or with anti-Rmp antiserum, as indicated beneath each section. IgG-reactive bands were disclosed with anti-human IgG or anti-mouse IgG conjugated to ALP. The two remaining sections were probed with the ALP conjugates alone, which served as background controls. All blots were developed for the same time. Black asterisks denote the location of Rmp. **(B)** IgG in the patient’s serum reacts against the gonococcal lipoproteins H.8 and Laz. Lysates of three *N. gonorrhoeae* strains (FA1090, WR220, and 15253) were separated on a 12% Bis-Tris gel, transferred to a membrane by western blotting, and probed with either the patient’s serum or anti-H.8/Laz mAb 2-8C-4-1. The locations of H.8 and Laz are indicated by red and blue asterisks, respectively. **(C)** Reactivity of IgG in patient’s serum with *N. meningitidis* H.8 and Laz lipoproteins. Western blotting of lysates of *N. meningitidis* strain MC58 and its isogenic H.8 and Laz deletion mutants was performed as described in B. Samples were run until the 15 kDa marker was at the bottom of the gel to better separate bands in close proximity to the H.8 band. The positions of H.8 and Laz are indicated by red and blue asterisks, respectively. A band that migrates just above H.8 is seen in the blot probed with the patient’s serum in the MC58 ΔH.8 lane. MW, molecular weight marker (kDa); def., deficient; mAb, monoclonal antibody. Source data are available for this figure: SourceData F4.

Further, we performed whole-genome sequencing on the bacterial isolate, which revealed that the strain possessed the *porB1b* allele with 100% amino acid identity to the porB (NEIS2020) allele 5325 in PubMLST (Fig. S1). We considered whether the patient had circulating antibodies against neisserial proteins known to be targets for blocking antibodies, so called because they can block killing by otherwise bactericidal antibodies. Targets for blocking antibodies include reduction-modifiable protein (Rmp) on *N. gonorrhoeae*, and H.8 (Lip) and lipidated azurin (Laz) on *Neisseria meningitidis*. Serum collected 2 mo after his initial positive blood culture showed anti-Rmp that was present in the wild-type strain but absent in the Rmp deletion mutant strain (Fig. 4 A). As expected, several other prominent bands were noted, including bands adjacent to the 6.5 kDa marker, which was likely lipooligosaccharide, and one at ∼21 kDa, which was consistent with H.8. We have shown previously that human anti-H.8 antibodies can block killing of *N. meningitidis* by antibodies against a key group B meningococcal vaccine antigen called factor H (FH) binding protein (9). We then probed western blots of whole-cell lysates from three different gonococcal strains against the patient’s serum and, in parallel, with an anti-H.8 monoclonal antibody, which showed an identical binding pattern of the ∼21 kDa band in both blots, suggesting that this band was indeed H.8 (Fig. 4 B). We confirmed that the patient’s serum also reacted with H.8 and Laz on *N. meningitidis*, where binding specificity was confirmed using isogenic mutants that lacked either H.8 or Laz (Fig. 4 C).

## Discussion

We herein report the first case of DGI identified in a patient with complement FI deficiency. FI deficiency has not been previously reported in disseminated gonococcemia, albeit the role of other complement system components in preventing disseminated neisserial infections has long been emphasized in prior studies (10, 11, 12). Complement deficiencies may be associated with inborn errors of immunity that manifest with phenotypic heterogeneity (13). FI controls the activation of the alternative complement pathway, so FI deficiency leads to increased production of C3b and secondary consumptive C3 insufficiency. Our patient presented with DGI and prior history of *N. meningitidis* infection. Additionally, he had similar presentations of rash and arthralgia in 2021 and 2023 that were attributed to IgA vasculitis, which, however, in light of his new DGI diagnosis raise suspicion for undiagnosed DGI or other undiagnosed bacterial infections resembling serum sickness; empiric broad-spectrum antibiotics that he received during these past admissions could have contributed to resolution. DGI can manifest as a triad of migratory polyarthritis, skin lesions, and tenosynovitis, or less commonly as purulent arthritis, or even as isolated rash (14). Our work highlights the importance of FI deficiency in recurrent neisserial infections. It also underscores the significance of maintaining high suspicion for FI functional defects in cases of low levels of components of the alternative complement pathway and terminal complement components, as this can be the result of consumptive complementopathy and be characterized by fever, rash, arthralgia, hematuria, and proteinuria.

The complement FI mutation carried by the patient is extremely rare at 0.006% (variant rs121964913). Specifically, our patient was homozygous for a nonsense mutation at codon 1420 with a nucleotide substitution from cytosine to thymine resulting in a stop codon in the C-terminal domain in the serine proteinase region (15). This mutation has been reported in the literature in the context of atypical hemolytic uremic syndrome (HUS), also known as complement-mediated HUS, (15) and recently has been reported as being significantly enriched with age-related macular degeneration (16). This is the first report of an association between this particular FI mutation with neisserial infection. Complement FI deficiency can present with diverse phenotypes depending on whether the deficiency is complete or partial. Complete FI deficiency has been linked with immunodeficiency and higher risk for infections with encapsulated bacteria, although these are mostly cases caused by *N. meningitidis*, *Streptococcus pneumoniae*, and *Haemophilus influenzae*; no case of DGI has been proven to be associated with this deficiency until now. Systemic lupus erythematosus–like manifestations, vasculitis, and central nervous system inflammation (deficiency of FI with cerebral inflammation or DEFICIT syndrome) of various degrees have also been reported (17, 18, 19, 20). Partial FI deficiency has been mostly related to complement-mediated HUS (19).

Interestingly, in our case, a low but detectable level of FI was reported by the reference laboratory. This was unexpected given that attempts by others to express FI R456X in cell culture did not result in any detectable secreted FI (7). We initially suspected that the nephelometry-based assay for FI was detecting the FI heavy chain in the absence of the light chain (protease domain) and results could be misleading depending on the specificity of the anti-FI antibody. However, neither the heavy nor the light chain of FI was detected in the patient’s serum using the same anti-FI antibody used by the clinical laboratory for nephelometry, so the lower limit of detection for the nephelometry may have been inaccurate. Genetic testing was critical to precisely pinpoint the complement pathway defect in this patient.

The patient’s isolate expressed the PorB.1B major outer membrane porin protein. The *N*. *gonorrhoeae* outer membrane is rich in PorB protein that plays a fundamental role in the ability of neisserial strains to disseminate. *N. gonorrhoeae* express a single PorB allele that belongs to either the PorB.1A or PorB.1B class. PorB comprises 16 antiparallel transmembrane ß-strands with 8 surface extracellular loops (8). Almost all of the PorB sequence variability resides in the extracellular loops. Expression of PorB.1A, called serogroup WI in an older classification scheme, is strongly associated with DGI (21, 22). PorB.1A-expressing strains are about 20 times more likely than PorB.1B-expressing strains to cause DGI (21, 23). Properties of PorB.1A strains that promote dissemination include a greater ability to traverse cells (24, 25) and evade killing by complement by virtue of their ability to bind to complement inhibitors, such as FH and C4b-binding protein (C4BP) (26, 27). Some PorB.1B isolates also bind FH and C4BP, are highly resistant to complement, and have been isolated with DGI (21, 26). Unfortunately, the frozen bacterial stocks we obtained from the clinical microbiology laboratory were nonviable; therefore, we could not assess this strain’s ability to evade complement. In almost every study, PorB.1B isolates outnumber PorB.1A strains, when all manifestations of gonorrhea are considered. It is not known whether *N. gonorrhoeae* strains from persons with complement deficiencies, in whose sera otherwise complement-sensitive strains would survive, would show a distribution similar to that seen in the general population (i.e., a PorB.1B bias). We speculate that complement consumption due to FI deficiency in this patient may have contributed to the ability of his PorB.1B to disseminate. Since PorB.1B was sequenced from the strain isolated from this patient, it was felt that this was less likely the key factor contributing to DGI, but rather dissemination of *N. gonorrhoeae* in this patient was primarily related to his FI deficiency.

Almost four decades ago, Rice and colleagues showed that antibodies against Rmp could block killing of gonococci by bactericidal sera (28, 29). Although the mechanism of blocking has not been fully elucidated, anti-Rmp antibodies are believed to direct complement components to nonbactericidal sites on the bacterial membrane (29). Human sera that contain antibodies directed against the AAEAP pentapeptide repeat motifs present in H.8 (Lip) and Laz proteins inhibit classical pathway activation by reducing C4b deposition, thereby blocking killing by select antibodies, such as those directed against meningococcal FH binding protein (9). Collectively, these data reiterate how the pathogenic Neisseriae have evolved to subvert host immune responses. Rmp and H.8 are abundantly expressed, have conserved amino acid sequences, and elicit antibodies in patients with neisserial infection (28, 30). H.8 possesses palmitic acid (C_16_ fatty acid, the typical Pam_3_Cys bacterial lipoprotein) and a C_8_-C_10_ fatty acid component that stimulate Toll-like receptor 2 (31), which may be responsible for its immunogenicity. Thus, antibodies against Rmp and H.8 could further predispose this patient to recurrent invasive gonococcal and meningococcal disease, respectively.

We recommended that the patient and his family members undergo genetic counseling. We also requested the patient to engage with his rheumatologist and to consider referral to immunology, nephrology, and ophthalmology specialists given the risk for complement-mediated HUS and age-related macular degeneration. However, the patient reported feeling well, canceled subsequent appointments, and has been lost to follow-up. The Advisory Committee on Immunization Practices of the United States Centers for Disease Control and Prevention recommends routine meningococcal vaccination for individuals with complement deficiencies (32). This includes both the quadrivalent MenACWY and group B meningococcal vaccines. Despite the potential for reduced vaccine efficacy in these patients, vaccination is still recommended due to the possible benefit in reducing the risk of infection. Additionally, vaccination against *Streptococcus pneumoniae* and *Haemophilus influenzae* type b is also advised (4, 32, 33). For patients with complement deficiencies, particularly those with terminal pathway defects, prophylactic antibiotics are often recommended. Penicillin or other suitable antibiotics may be used to provide ongoing protection against *N*. *meningitidis*. However, such prophylaxis is not established practice for patients with *N*. *gonorrhoeae* infections (32, 34, 35).

In conclusion, our work reveals the first reported patient with risk for DGI and recurrent neisserial infections linked to FI deficiency. Typically, this risk has been associated with deficiency of other complement components, while the mutation identified in our patient leading to the absence of FI protein has been associated with other disease manifestations (complement-mediated HUS and macular degeneration). Identification of this rare mutation in patients with low CH50 may aid in taking additional steps to prevent further neisserial infections such as administration of prophylactic antibiotics and vaccinations. Studies examining antibodies directed against targets for blocking antibodies such as Rmp and H.8 may identify individuals at heightened risk for recurrent, invasive neisserial infections in persons with complement defects, including FI deficiency.

## Materials and methods

### Sera and antibodies

NHS was prepared by pooling sera obtained from five volunteers with no known immunodeficiencies. FI-depleted serum (Cat# A338) was purchased from Complement Technology, Inc. Remnant serum following measurement of individual complement components at Cincinnati Children’s Hospital was sent to UMass Chan Medical School for western blotting. Goat antiserum to human FI was from Complement Technology, Inc. (Cat# A238). Rabbit anti-goat IgG (whole molecule)–alkaline phosphatase (ALP) antibody (Cat# A7650) and goat anti-mouse IgG–ALP antibody (Cat# A9316) were from MilliporeSigma. Goat anti-human IgG (Fcγ fragment–specific)–ALP antibody (Cat# 109-055-008) was from Jackson ImmunoResearch. Mouse antiserum against Rmp was raised by immunizing mice with a peptide fragment of Rmp (amino acid sequence CWKNAYFDKAS; synthesized by GenScript) conjugated to cross-reactive material (CRM; conjugation performed by Fina Biosolutions). 6-wk-old BALB/c mice were immunized intramuscularly at 0, 2, and 4 wk with 20 µg of CRM-Rmp peptide adjuvanted with AddaVax (Cat# vac-adx-10; InvivoGen). Anti-Rmp IgG was purified from immune sera by affinity chromatography over protein A/G agarose and used for western blotting at a concentration of 1 µg/ml. Monoclonal antibodies against lipoprotein H.8 and lapidated azurin 2-8C-4-1 have been described (36).

### Bacterial strains

*N. gonorrhoeae* strains FA1090 (37), WR220 (38) and 15253 (39), and meningococcal strain MC58 (40) have been described. FA1090 ΔRmp was created by insertional activation of *rmp* with the ErmR cassette using homologous recombination. Isogenic mutants of meningococcal strain MC58 that lack the lipoproteins H.8 (MC58 ΔH.8) or lapidated azurin (MC58 ΔLaz) have been described (9).

### Bacterial whole-genome sequencing

Genomic DNA was isolated from bacteria using the Qiagen DNeasy Blood & Tissue Kit (Cat# 69504) according to the manufacturer’s instructions. Genomic sequencing was performed using Nextera XT DNA library preparation kits followed by 150 nt paired-end sequencing on an Illumina NextSeq 2000 sequencing system. The genome was assembled and annotated using shovill v1.1.0 (https://github.com/tseemann/shovill) and prokka v1.14.6 (41), respectively. The sequence reads and assembly can be found under BioProject ID PRJNA1258989 at https://www.ncbi.nlm.nih.gov/bioproject/1258989.

### Western blotting

NHS, FI-depleted serum, and the patient’s serum were diluted at 1:100 in phosphate-buffered saline (PBS) and 4× lithium dodecyl sulfate sample buffer (either with or without ß-mercaptoethanol). Serum proteins were separated on a 4–12% NuPAGE Bis-Tris Mini Protein Gel (Cat# NP0322; Thermo Fisher Scientific) with 4-morpholineethanesulfonic acid sodium dodecyl sulfate (MES SDS) running buffer (Cat# B0002; Thermo Fisher Scientific). Goat anti-human FI and rabbit anti-goat IgG–ALP antibody were diluted 1:1,000 in PBS/Tween-20 for incubation for 1 h at room temperature. The blot was developed with 5-bromo-4-chloro-3-indolyl-phosphate/nitro blue tetrazolium. In a separate assay, the patient’s serum was diluted at 1:500 in PBS/Tween-20 for use as a probe against bacterial whole-cell lysates. Blots were incubated with serum or mouse anti-RMP for 1 h at room temperature. Secondary antibodies included anti-human IgG–ALP and anti-mouse IgG–ALP.

### Online supplemental material

The annotated PorB sequence of the patient’s *N. gonorrhoeae* isolate is available as Fig. S1.

## Supplementary Material

SourceData F3 is the source file for Fig. 3.

SourceData F4 is the source file for Fig. 4.

## Data Availability

All data are available in the article and supplementary materials.
