# Supplementary figures and images for: Disseminated gonococcal infection secondary to a rare homozygous mutation resulting in complement factor I deficiency
Source: J Hum Immun. 2025 Aug 18;1(3):e20250088. doi: 10.70962/jhi.20250088 (PMC12829745; doi:10.70962/jhi.20250088)

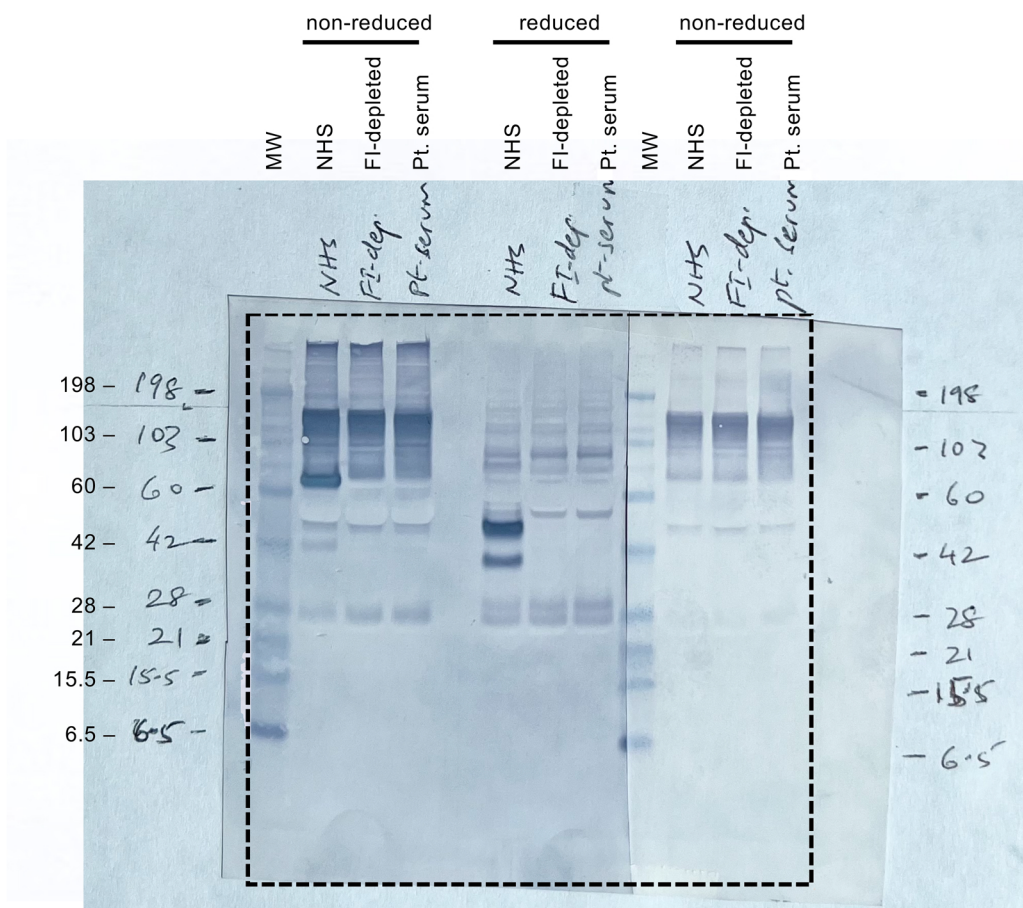

Uncropped Figure 3

Supplement: SourceData F3 — is the source file for Fig. 3. [file jhi_20250088_sourcedataf3.pdf]
