# Supplementary material for: Disseminated gonococcal infection secondary to a rare homozygous mutation resulting in complement factor I deficiency
Source: J Hum Immun. 2025 Aug 18;1(3):e20250088. doi: 10.70962/jhi.20250088 (PMC12829745; doi:10.70962/jhi.20250088)
Supplement: SourceData F4 — is the source file for Fig. 4. [file jhi_20250088_sourcedataf4.pdf]

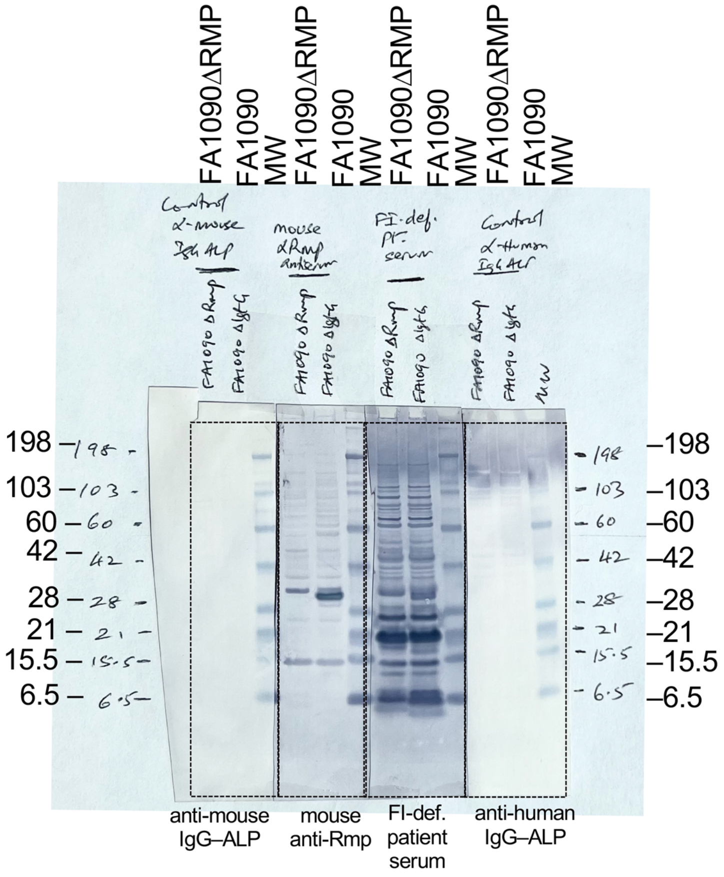

Uncropped Figure 4A
(Note: blot is flipped in Figure 4A)

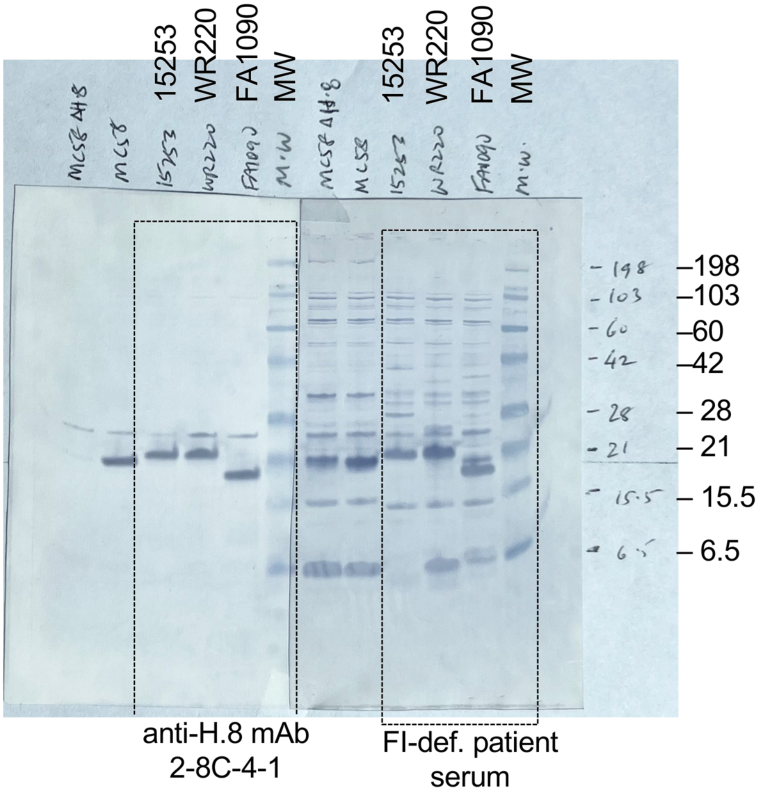

Uncropped Figure 4B
(Note: blot is flipped in Figure 4B)

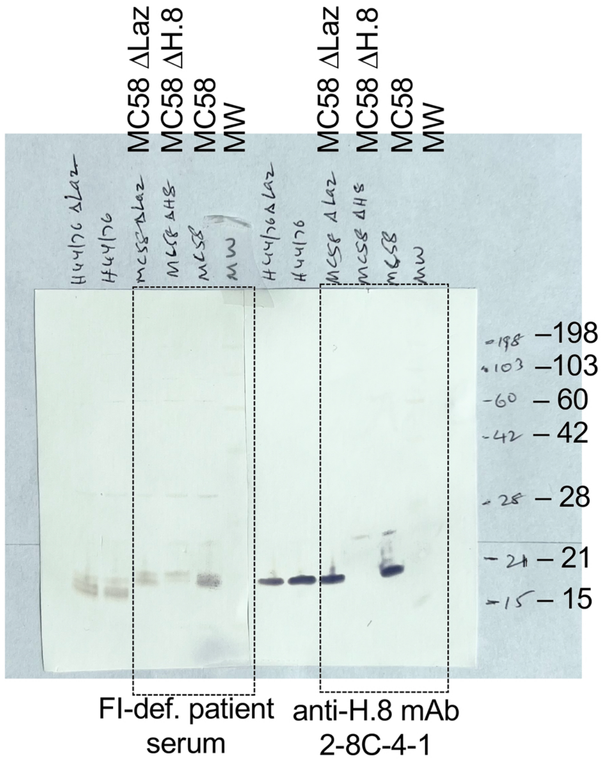

Uncropped Figure 4C

Source Data for Figure 4
